# Design of Multifunctional SC-PLA Pesticide Carrier System and Study of Controlled-Release Performance

**DOI:** 10.3390/ma19030492

**Published:** 2026-01-26

**Authors:** Xuanxuan Wang, Ruizhe Wang, Dongxia Han, Yaling Zhou, Qinwei Gao

**Affiliations:** 1 College of Chemical Engineering, Nanjing Forestry University, Nanjing 210037, China; 15098299627@njfu.edu.cn (X.W.); wrz_njfu@njfu.edu.cn (R.W.); 8240210157@njfu.edu.cn (D.H.); 18609617692@163.com (Y.Z.); 2 Jiangsu Key Laboratory for the Chemistry and Utilization of Agricultural and Forest Biomass, Nanjing Forestry University, Nanjing 210037, China

**Keywords:** polylactic acid, avermectin, nanospheres, pH, stereocomposite crystals

## Abstract

To construct a high-performance avermectin (Avm) carrier system, this study utilized the advantages of stereocomplex (SC) crystal formation between poly (L-lactic acid) (PLLA) and poly (D-lactic acid) (PDLA) to prepare Avm-loaded stereocomplex polylactic acid (SC-PLA) nanoformulations via the emulsion solvent evaporation method. The results showed the successful formation of SC-PLA after introducing PDLA into the PLLA matrix, and the influence of SC-PLA crystallinity enabled the fabrication of tunable Avm@SC-PLA nanospheres with a regular spherical morphology. Avm@SC-PLA exhibited controlled release characteristics and possessed pH-responsive properties with specific release behaviors under pH 5.5, 7.4, and 8.0 conditions. The Avm@SC-PLA sustained-release nano system had a series of advantages, including controllable particle size, efficient drug loading, excellent sustained-release performance, good UV-shielding ability, high stability, favorable spreadability, and strong affinity for different leaves. In conclusion, the Avm@SC-PLA nanoformulation not only achieves effective loading and stable encapsulation of Avm but also possesses good structural stability and environmental responsiveness. It provides a novel PLA-based carrier strategy for the efficient delivery of Avm and holds potential application value in the pesticide and pharmaceutical fields.

## 1. Introduction

As a foundational industry of the national economy, the stable development of agriculture bears direct implications for national strategic security and social livelihood security [1]. Within this framework, grain production security constitutes the core link of the agricultural system, with its safeguarding mechanisms attracting extensive scholarly attention [2]. In modern agricultural production systems, chemical pesticides continue to play a pivotal role owing to their high-efficacy containment capacity against diseases and pests. Avermectin (Avm) and its derivatives, as a class of bioactive substances with a 16-membered macrocyclic lactone structure, have been widely used in the field of agricultural pest control due to their prominent environmental friendliness and target specificity [3,4,5,6]. However, the chemical stability of Avm is susceptible to factors such as ultraviolet radiation and extreme pH environments, which may lead to premature degradation of the drug substance [7]. Consequently, substantial research efforts have been devoted to developing sustained-release systems to achieve controlled delivery efficacy of Avm.

In the relevant research on drug delivery systems, polylactic acid (PLA) exhibits multiple superior properties that distinguish it from traditional polymers [7,8,9,10]. Its raw materials for PLA production are widely available from the fermentation of starch. The internal factors of PLA materials themselves, such as structure, molecular weight, crystallinity, and hydrophilicity–hydrophobicity, determine that PLA materials have excellent degradability, good processability, and high mechanical strength. This unique combination of material properties enables PLA to occupy an important position in applications such as orthopedic fixation devices, surgical sutures, and drug sustained-release systems [11]. Wang [12] studied monodisperse nanoscale PLA sustained-release nanospheres with the anticancer drug hydroxycamptothecin, which laid the foundation for the subsequent preparation of implants. In recent years, stereocomplex polylactic acid (SC-PLA) has attracted extensive research attention in the field of biomedical materials due to its unique physicochemical properties [13]. The Ikada [14] research group discovered that molecular-level blending of poly (L-lactic acid) (PLLA) and poly (D-lactic acid) (PDLA) yields stereocomplexes with highly ordered crystalline structures. This landmark finding not only pioneered new research directions for PLA materials but also established the theoretical foundation for subsequent performance optimization and application development of SC-PLA. Compared with neat PLLA, SC-PLA exhibits significant advantages in thermal stability, resistance to solvent erosion, and mechanical properties, which are mainly attributed to the unique molecular arrangement and lattice structure characteristics of its stereocomplex crystals [15,16,17]. Due to the strong intermolecular interactions between the enantiotopic chains of PLLA-PDLA pairs, SC-PLA has a higher melting temperature, better mechanical properties, and stronger hydrolysis resistance [18].

To optimize the deposition uniformity of pesticide active ingredients on plant leaf surfaces, researchers have developed a new delivery system based on nanocarriers [19]. This technology utilizes the unique size effect (with a particle size range of 50–500 nm) and high specific surface area characteristics of nanomaterials, significantly enhancing the utilization rate of active ingredients [20]. Its core mechanism involves encapsulating pesticide active molecules with degradable polymer materials (such as SC-PLA) to form nanospheres with sustained-release functionality, thereby achieving precise spatiotemporal controlled-release effects [21]. Additionally, Bai et al. [22] demonstrated that PDLA can function as both nucleating agent and rheology modifier for PLLA. The formation of SC-PLA alters the morphological evolution and self-assembly behavior of PLLA/PDLA blends. Consequently, SC-PLA nanospheres, as polymeric carriers for controlled delivery systems, enable precise modulation of parameters including particle size, morphology, composition, and polymer molecular weight. This tunability optimizes their release kinetics, biodegradability, and mechanical properties [23,24,25]. These carrier materials exhibit non-toxic and biocompatible characteristics, demonstrate chemical inertness toward encapsulated drugs, and provide controllable regulation over drug release rate. Yang et al. [26] designed and fabricated a PLA/magnetic nanoparticle (MNP) composite system. Increasing MNP contents promoted the surface crystallization and enhanced the wettability of PLA. This modification enables controlled drug release by regulating PLA hydrolysis kinetics. Haers et al. [27] demonstrated that modulating the PLLA/PDLA ratio within the PLA blends significantly enhances the mechanical properties of PLA, enabling successful fabrication of fracture fixation screws and metallic stabilization plates. Post-implantation, these scaffolds undergo hydrolytic degradation over time without requiring enzymatic catalysis or surgical removal. This research provided prospective foundational evidence for biomechanical stability modeling in bilateral jaw osteotomy using biodegradable fixation systems [28]. The stereocomplex crystals formed by PLLA and PDLA exhibit significantly higher crystallinity and thermal stability than the homocrystal of single-component PLA. This enables the nanospheres to resist deformation during storage, processing, or in environmental media, with more controllable degradation rates. Meanwhile, SC-PLA inherits the biodegradability and biosafety of PLA: it can degrade into non-toxic lactic acid in vivo, which then participates in metabolism, and can naturally degrade in the environment, avoiding residual pollution. These properties make SC-PLA suitable for the green requirements in both pharmaceutical and pesticide fields. Therefore, when SC-PLA nanospheres are used as drug carriers, it is feasible to consider regulating drug release by modifying these influencing factors to improve therapeutic efficacy or achieve some therapeutic goals with special requirements [29].

This work establishes a programmable crystallization engineering strategy for SC-PLA to architect novel Avm nano-delivery systems with controlled release profiles. The high-speed shearing method was employed in this study, and SC-PLA nanospheres were successfully fabricated by introducing different proportions of PDLA into the PLLA solution. By modulating the mass ratio of PLLA to PDLA, the particle formation process was optimized, and the physical state of Avm encapsulated in the nanospheres was systematically evaluated. In addition, the drug delivery performance, system stability, and ultraviolet (UV) degradation resistance of the nanoparticles were comprehensively determined and analyzed. In summary, by precisely modulating the crystallinity, porosity, and drug release kinetic parameters of SC-PLA nanospheres, this nanomaterial achieves efficient delivery and stable protection of Avm, providing a novel biology-based solution for the development of eco-friendly pesticide formulations.

## 2. Materials and Methods

### 2.1. Materials

Low-molecular-weight poly (D-lactic acid) (PDLA, Mn = 6700 g/mol, Ð = 1.10) and the commercial PLLA (product number: LX575; Mn = 100,000 g/mol; Ð = 1.6) were purchased from Jinan Daigang Biomaterial Co., Ltd. (Jinan, China). The commercial avermectin (Avm) (18 g/L^−1^) was purchased from Shanghai Shengnong Pesticide Co., Ltd. (Shanghai, China). Polyvinyl alcohol (PVA) with a Mw of 30,000–70,000 and a hydrolysis of 87–89% was purchased from Aladdin (Shanghai, China). Dichloromethane (DCM) and all other chemicals were purchased from Nanjing Chemical Reagent Co., Ltd. (Nanjing, China).

### 2.2. Preparation of SC-PLA Nanospheres

The PLLA and SC-PLA nanospheres with controlled stereocomplexation were prepared by varying PLLA/PDLA ratios through a high-speed shearing method. PLLA and PDLA were introduced into 50 mL of DCM solution, with the mass ratios of PLLA to PDLA being 10:0, 7:3, 3:7 and 1:9, respectively. Subsequently, the organic phase was added dropwise at a constant rate of 1 mL/min into a 1.0% (*w*/*v*) PVA aqueous solution. The mixture was first subjected to high-speed shearing treatment for 5 min at room temperature, followed by magnetic stirring at 1000 r/min for 6 h to maximize the volatilization of DCM [30].

The resulting mixture was centrifuged and washed three times with deionized water to remove residual PVA. The above dispersion was centrifuged again to eliminate large-sized aggregates and undissolved materials in the system. Finally, the obtained nanoparticles were pre-frozen in an ultra-low-temperature refrigerator at −40 °C for 12 h, then immediately transferred to a vacuum freeze dryer for lyophilization. The dried nanoparticle powder was obtained and reserved for subsequent solid-state characterization.

**Figure 1:**
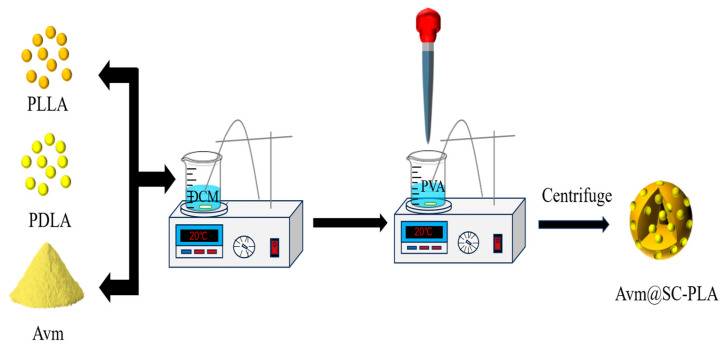
Synthetic illustration showing the preparation of Avm@SC-PLA.

The preparation of Avm-loaded SC-PLA nanospheres followed the same procedure as blank nanospheres, according to the process shown in Figure 1. During the fabrication of SC-PLA nanospheres with varying ratios, a predetermined amount of Avm was co-dissolved in 50 mL of DCM. The following steps were identical to the previous description.

### 2.3. Characterizations

#### 2.3.1. Differential Scanning Calorimetry (DSC) and X-Ray Diffraction (XRD)

The non-isothermal crystallization kinetics of the synthesized copolymers and blends were characterized by DSC measurements on a NETZSCH DSC-200F3 differential scanning calorimeter (NETZSCH-Geratebau GmbH, Selb, Germany). The first heating was from 25 °C to 250 °C at a heating rate of 20 °C/min to eliminate the thermal history of the blends. The sample was kept isothermal for 5 min, then cooled from 250 °C to 20 °C at a cooling rate of 15 °C/min and a nitrogen gas flow of 50 mL/min, and finally reheated to 250 °C again at a heating rate of 10 °C/min. The crystallinity of the nanospheres was analyzed using the following equation:

(1) $$X_{c}\;=\;\frac{{\text{∆}H}_{m}}{\;{\text{∆}H}_{m}^{0}}\;\times \;100\%$$

∆H_m_ is the melting enthalpy of homocrystals (HCs) or SC crystallites and ${\text{∆}H}_{m}^{0}$ is the melting enthalpy of 100% HC or SC crystalline PLA correspondingly. For HC crystallites, ${\text{∆}H}_{m}^{0}\;=\;93\;J/g,$ and for SC crystallites, ${\text{∆}H}_{m}^{0}\;=\;142\;J/g$.

The crystal structure of the synthesized copolymers and blends was analyzed by XRD on a Rigaku XRD Ultima IV instrument (Rigaku Corporation, Tokyo, Japan) with a D/teX-Ultra using Ni-filtered Cu Kα radiation (λ = 0.154 nm), operated at 40 kV and 30 mA. The sample was step-scanned from 5° to 35° at a 2θ scanning rate of 5°/min.

#### 2.3.2. Scanning Electron Microscope (SEM) and Transmission Electron Microscope (TEM)

To investigate the morphological characteristics of dried Avm@SC-PLA nanodrugs, this study employed a scanning electron microscope (SEM, Model Quanta 200, Hillsboro, OR, USA) for characterization and analysis. The dried Avm@SC-PLA materials were directly deposited on specimen stubs, subjected to vacuum gold-sputtering treatment, and then images were captured at different magnification levels. Additionally, a transmission electron microscope (TEM, Model JEM-1400, JEOL, Tokyo, Japan) was used to analyze the internal structure of the materials. Prior to TEM imaging, Avm@SC-PLA aqueous solutions were dropped onto carbon-coated copper grids, followed by drying at 25 °C, and an accelerating voltage of 180 kV was applied during the TEM testing process.

For transmittance measurement, the Avm@SC-PLA suspension was subjected to ultrasonic treatment to ensure uniform dispersion, followed by testing with a UV–visible spectrophotometer (UV-2450, Shimadzu Corporation, Kyoto, Japan) over the wavelength range of 200–800 nm. Fourier transform infrared (FTIR) spectroscopy analysis was performed using the KBr pellet method: lyophilized nanospheres were mixed with potassium bromide (KBr) and compressed into pellets. The measurements were conducted with an FTIR spectrometer (Nicolet 380, Thermo, Waltham, MA, USA) in the wavenumber range of 500–4000 cm^−1^ to analyze the chemical structural characteristics of the samples.

### 2.4. Measuring the Loading Capacity of Drug

High-efficiency pesticide formulations usually require high loading content and entrapment efficiency of pesticides in carriers to save time, manpower, and resources during the preparation process, as well as to avoid extensive use in the spraying process. The nanoparticle dispersion was centrifuged at 12,000 rpm for 30 min, and the supernatant was collected. The absorbance of free Avm@SC-PLA was measured at 245 nm using UV–visible spectrophotometry. The concentration of free Avm in the mixture was quantified against a pre-established calibration curve. Triplicate measurements were performed for each sample, with the arithmetic mean used for subsequent calculations. The encapsulation efficiency (EE) and loading capacity (LC) were then calculated using the following equations (Table S2) [31].

(2) $$EE\left(\%\right)=\frac{\mathrm{total}\;\mathrm{Avm}\;-\;\mathrm{Free}\;\mathrm{Avm}}{\mathrm{total}\;\mathrm{Avm}}\text{×}100\%$$

(3) $$LC\left(\%\right)=\frac{\mathrm{total}\;\mathrm{Avm}\;-\;\mathrm{Free}\;\mathrm{Avm}}{\mathrm{total}\;\mathrm{mass}\;\mathrm{of}\;\mathrm{nanoparticles}}\text{×}100\%$$

### 2.5. Deposition Rate Studies

To investigate foliar retention and leaching loss, uniformly sized leaves (11 cm × 4 cm) were ultrasonically cleaned to remove surface particulates, immersed in deionized water for 20 min with three rinsing cycles, and dried. Using distilled water as blank control, contact angles (CA) of formulated agents were measured on fresh leaf surfaces with a contact angle goniometer (Model JC2000C1, Shanghai Zhongchen Digtal Technology Apparatus Co., Ltd., Shanghai, China). Test solutions were deposited via precision microsyringe, with CA values quantified according to literature methods [32].

All experiments were conducted in triplicate, and statistical analysis was performed using IBM SPSS Statistics 27 software with one-way ANOVA to evaluate the data. A *p*-value of <0.05 was considered statistically significant.

For retention assays, newly cleaned leaves were weighed, mounted at a 45° inclination with forceps, and uniformly sprayed with 1.5 mL of sample solutions from a fixed distance. After 20 s of deposition, the leaves were reweighed to determine retained mass. Equation (4) can be used to calculate the deposition rate (D%) as follows:

(4) $$D\left(\%\right)=\frac{W_{2}\;-\;W_{1}}{W_{1}}\text{×}100\%$$

where W_1_ is the weight of the leaves before solution spraying and W_2_ is the weight of the leaves following solution spraying. For every experiment, three duplicates were set up.

### 2.6. Anti-Photodegradation of the Nanoparticles

Accurately weighed quantities of Abamectin Technical Material and Abamectin-loaded nanoparticles, each containing 20 mg of active ingredient, were transferred into UV-transparent polystyrene Petri dishes and subjected to continuous irradiation under a 250 W mercury-vapor UV lamp at 25.0 ± 0.5 °C. The samples were removed at 12 h, 24 h, 48 h, 60 h and 75 h, respectively, and measured using the UV spectrophotometry method, according to the standard curve equation, three times. The average values were determined, and the photolysis rates were calculated.

### 2.7. Stability Tests of Nanoparticles

The stability of the abamectin nanoformulation was tested through cold and hot storage [33]. The abamectin nanosuspension was aliquoted into clean brown vials, which were then hermetically sealed and stored separately in a refrigerator (0 °C), at room temperature (25 °C), and in a 55 °C incubator. After 14 days of storage, a Malvern Zetasizer Nano-ZS laser particle size analyzer (Worcestershire, UK) was used to determine the Z-average particle size and PDI of the abamectin nanoemulsions.

### 2.8. Drug Release Behavior

Instead of qualitative release behaviors for the traditional pesticide formulations, novel pesticide formulations with accurate release capability were desired for customized practical applications [34]. To investigate the effect of pH on the release behavior of Avm, Avm nano-delivery samples with different proportions were separately suspended in 5 mL of buffer solutions with pH values of 5.5, 7.4, and 8.0. Subsequently, the suspension was transferred into a dialysis bag; after sealing the dialysis bag, it was separately placed in brown flasks containing 95 mL of buffer solutions with the corresponding pH values. The flasks were then placed in an incubator shaker and incubated with shaking at 300 rpm under room temperature to ensure the consistency of the experimental environment across all pH groups. At predetermined time intervals (e.g., 1 h, 2 h, 4 h, 8 h, 12 h, 24 h, 50 h), 5.0 mL of solution was taken from the release medium of each pH group, and immediately replaced with 5.0 mL of fresh buffer solution with the corresponding pH value (to maintain a constant total volume of the release medium and ensure the accuracy of subsequent concentration calculations) [35]. By measuring the concentration of dissolved Avm in the release medium of each pH group at different time intervals, the Avm release rate of the nano-delivery samples under different pH conditions was calculated separately, and their pH-responsive release performance was further evaluated by comparison. The concentration of Avm was determined using a UV-vis spectrophotometer at a wavelength of 245 nm. To further verify the stability of the experimental results, the above experimental procedure was repeated three times in parallel under each of the aforementioned pH conditions. This ensures the reliability, repeatability, and statistical significance of the experimental data, and provides a scientific basis for clarifying the pH-responsive release law of the Avm nano-delivery system.

## 3. Results and Discussion

### 3.1. Preparation and Characterization of SC-PLA

**Figure 2:**
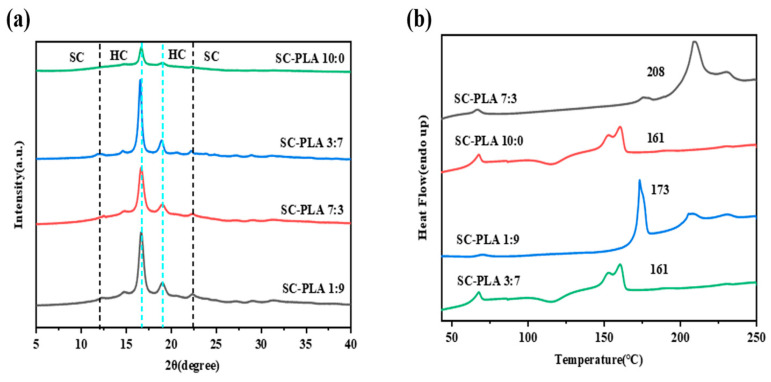
(**a**) XRD patterns and (**b**) DSC first heating curves of SC-PLA nanospheres.

The incorporation of PDLA into the PLLA matrix facilitates the formation of SC-PLA. Existing studies have verified that this process directly modulates the morphology of nanospheres (e.g., transforming from petal-like structures to regular spherical or golf ball-like shapes) as well as their pore structures (Figure S1) [36]. Meanwhile, the formation of SC-PLA exerts a significant influence on the crystallinity of nanospheres, and the variations in crystallinity are further correlated with key performance metrics of nanospheres, such as degradation rate, drug release efficiency, and permeability [30]. During the preparation process, as DCM evaporates, the distance between PLA chains shortens, allowing adjacent PLLA and PDLA chains to aggregate and form SC-PLA crystals. Therefore, the crystal form and crystallinity of PLA are undoubtedly core factors regulating the properties of nanospheres. In this study, the emulsion solvent evaporation method was employed to prepare nanospheres for encapsulating Avm. XRD and DSC were used to characterize the crystal structure properties (Table S1). As illustrated in Figure 2a, the PLLA nanospheres exhibit three typical characteristic diffraction peaks at 17°, 19°, and 22.5°, which reflect the homogeneous crystallization behavior of PLA. Figure 2b shows that the PLLA nanospheres exhibit a broad cold crystallization peak, and the melting peak at approximately 160 °C corresponds to the melting process of its high-crystallinity (HC) crystals (HC-PLLA). After the introduction of PDLA, the intensity of the HC-PLLA melting peak decreases, and a new melting peak appears at around 210 °C, indicating the formation of SC-PLA.

### 3.2. Synthesis and Analysis of Avm@SC-PLA Nanodrugs

**Figure 3:**
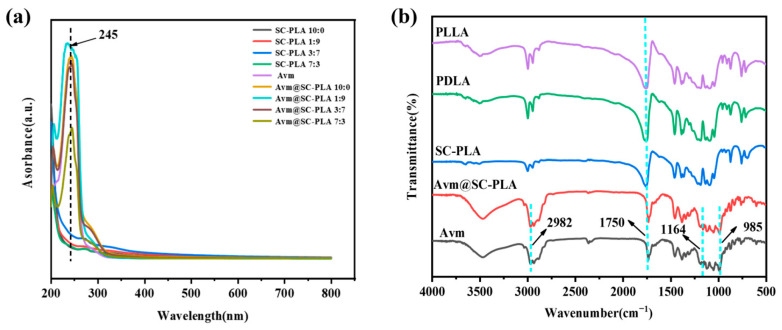
Characterizations of nanoparticles and nanodrugs. (**a**) UA-vis curves of PLLA, PDLA, SC-PLA, Avm@SC-PLA, and Avm. (**b**) FTIR curves of PLLA, SC-PLA 1:9, SC-PLA 3:7, SC-PLA 7:3, Avm@PLLA, Avm@SC-PLA 1:9, Avm@SC-PLA 3:7, Avm@SC-PLA 7:3, and Avm.

To further verify the effective preparation and successful drug loading of Avm@SC-PLA, this study employed FTIR and UV-Vis for multidimensional characterization. UV-vis spectroscopy results showed that Avm@SC-PLA exhibited an obvious characteristic absorption peak at 245 nm, which was completely consistent with the characteristic absorption peak of the Avm standard (Figure 3a). In contrast, no obvious absorption signal was observed for blank SC-PLA particles at this wavelength. This directly confirmed that Avm had been successfully loaded onto the SC-PLA carrier, and no significant chemical structure change of the drug occurred in the carrier matrix. FTIR results showed that both PLLA and PDLA exhibited obvious C=O stretching vibration bands at 1750 cm^−1^; the differences mainly lay in slight variations in peak intensity and position [37]. The C=O peak in SC-PLA showed a red shift, indicating that the C=O groups were affected by hydrogen bonding or crystal packing, and that there were interactions between carbonyl groups. Avm@SC-PLA not only completely retained the characteristic vibrational bands of ester groups (-C=O) and ether bonds (C-O-C) from blank SC-PLA but also exhibited a new characteristic vibrational band at 3400 cm^−1^, which corresponded to the hydroxyl groups (-OH) of Avm (Figure 3b).

**Figure 4:**
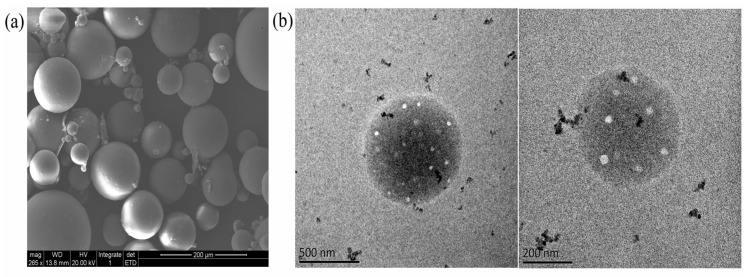
Microscopic morphology of nanoparticles and nanodrugs. (**a**) SEM images of Avm@SC-PLA. (**b**) TEM images of Avm@SC-PLA.

The high magnification TEM and SEM images showed that the SC-PLA particles had a smooth surface, a uniform internal structure, no obvious impurities or agglomeration, and an overall regular spherical morphology with a homogeneous particle size distribution and no significant size variation (Figure 4). This excellent micromorphological feature is closely related to the stereoregular arrangement of SC-PLA molecular chains—the dense crystalline structure formed by stereocomplexation provides crucial structural support for the uniform formation of the particles. Combining the above characterization results, it can be fully confirmed that Avm was successfully loaded onto the SC-PLA carrier, and the Avm@SC-PLA nanospheres possess good structural stability.

### 3.3. Contact Angle Test of Nanoparticles and Drug Carrier Deposition Characteristics

**Figure 5:**
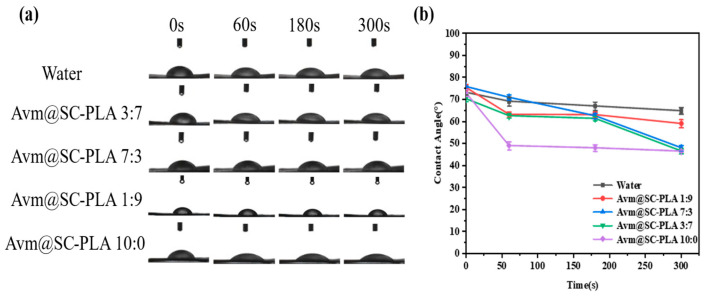
Contact angle test. (**a**) Images of control, water, Avm@SC-PLA 10:0, Avm@SC-PLA 1:9, Avm@SC-PLA 3:7, and Avm@SC-PLA 7:3 contact angles on a leaf surface. (**b**) Contact angle data comparison images for control, water, Avm@SC-PLA 10:0, Avm@SC-PLA 1:9, Avm@SC-PLA 3:7, and Avm@SC-PLA 7:3.

Enhancing the wetting ability and adhesion performance of drug droplets on plant leaf surfaces is a key means to improve pesticide deposition and utilization efficiency [38]. In contact angle measurement experiments, the wetting degree of leaves can be quantitatively characterized by the CA between leaves and the drug solution [39]. The adhesion efficiency of nanocarrier systems to plant leaves plays a core role in reducing drug fluidity and optimizing application effects [40]. The efficient deposition of pesticide formulations on crop leaf surfaces is of great significance for reducing losses and improving utilization efficiency. To investigate the wetting characteristics of Avm@SC-PLA nanoparticles with different proportions on rice leaf surfaces, we studied the wettability of the nanoformulations on rice leaves through contact angle tests (Figure 5a). To investigate the effects of PDLA content and Avm loading on the wettability of the SC-PLA system, the CA of deionized water was used as the blank control (Figure 5b). With the increase in the proportion of the PDLA component, the crystallinity of the SC-PLA material showed a synchronous upward trend, accompanied by enhanced surface hydrophobicity and a significant increase in the corresponding contact angle value. After Avm drug molecules were loaded onto the SC-PLA carrier, the introduction of drug molecules and the slight structural rearrangement on the carrier surface increased the number of hydrophilic sites on the material surface. Consequently, the nanoparticles did not exhibit ultra-strong hydrophobicity, which in turn optimized their wetting compatibility with plant leaf surfaces. In 300 s, the CA of Avm@SC-PLA nanomedicine with a PLLA:PDLA mass ratio of 10:0 decreased from 70.63 ± 0.3° to 46.14 ± 0.2°; under the same conditions, that of Avm@SC-PLA nanomedicine with higher crystallinity (PLLA:PDLA mass ratio of 1:9) dropped from 76.83 ± 0.4° to 59.67 ± 0.3°; the contact angle of Avm@SC-PLA nanomedicine with a PLLA:PDLA mass ratio of 3:7 fell from 73.36 ± 0.4° to 47.01 ± 0.3°; and that of deionized water decreased from 73.36 ± 0.3° to 66.47 ± 0.2°. Among all tested samples, the Avm@SC-PLA nanomedicine with a 10:0 ratio exhibited the lowest final contact angle and the most significant dynamic decrease. This result indicates that compared with deionized water and Avm@SC-PLA nanoparticles with higher crystallinity, those with lower crystallinity showed superior wettability and adhesion efficiency on rice leaf surfaces. They can effectively expand the contact area between the pesticide and the leaf surface, and promote the adsorption and deposition of active ingredients, thereby improving pesticide utilization efficiency and providing important support for optimizing its field application effect [41].

**Figure 6:**
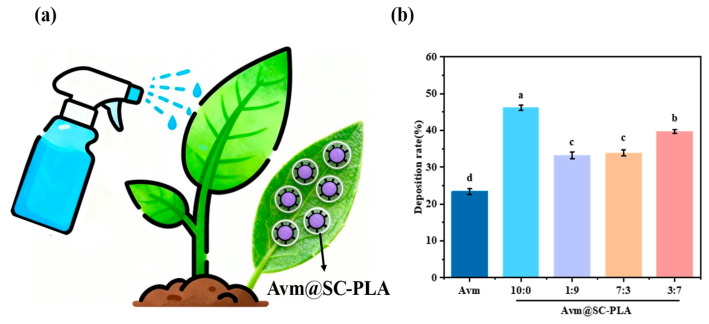
Retention capacity. (**a**) Schematic diagram of leaf spraying. (**b**) Comparison of data after spraying with water, Avm, Avm@SC-PLA 10:0, Avm@SC-PLA 1:9, Avm@SC-PLA 3:7, and Avm@SC-PLA 7:3. Different letters (a, b, c, d) indicate significant differences among groups (*p* < 0.05).

The retention degree of pesticides on plant leaf surfaces significantly impacts their actual efficacy after application [42]. Therefore, enhancing the deposition efficiency and adhesion properties of the drug carrier is a key approach to improving the retention capacity of pesticides on leaf surfaces [43]. Deposition experiments were conducted to evaluate the retention rate of the Avm@SC-PLA synthesized fungicide, and it was found that its optimization could significantly improve the retention effect of pesticides on plant surfaces. A spray can was used to sequentially spray Avm, Avm@SC-PLA with a 10:0 ratio, Avm@SC-PLA with a 1:9 mass ratio, Avm@SC-PLA with a 3:7 mass ratio, and Avm@SC-PLA with a 7:3 mass ratio onto the leaves at the same distance. The weight of the leaves was recorded, and the experiment was repeated three times. The results of the leaf retention rate test showed that the leaf retention rate of free Avm was 23.15%. For the drug-loaded Avm@SC-PLA nanoparticles with mass ratios of PLLA to PDLA of 10:0, 7:3, 3:7, and 1:9, their leaf retention rates were 45.62%, 33.43%, 39.75%, and 32.92%, respectively (Figure 6). These results indicate that compared to free Avm, the Avm@SC-PLA at a ratio of 10:0 exhibits higher leaf retention capability, which is consistent with the observed leaf contact angle data. This suggests that using Avm@SC-PLA at different ratios can significantly enhance the adhesion ability of pesticides on leaf surfaces by optimizing the carrier structure and interfacial interactions.

### 3.4. Photolysis Property of Avermectin (Avm) Nanoformulation

**Figure 7:**
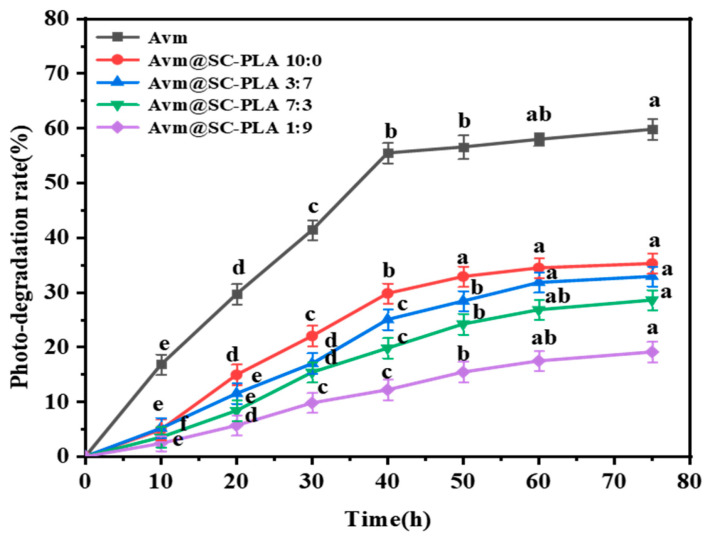
Responsive photodegradation curves of active Avm, Avm@SC-PLA 10:0, Avm@SC-PLA 1:9, Avm@SC-PLA 3:7, and Avm@SC-PLA 7:3 versus irradiated time (UV light). Values marked with different superscript letters (a, b, c, d, e) denote statistically significant differences among the groups (*p* < 0.05).

As a widely used fungicide, Avm is highly prone to degradation when exposed to ultraviolet (UV) light, which greatly limits the durability of its field application effects [44]. To address this issue, optimizing the formulation through encapsulation technology has emerged as an effective technical solution to enhance its photostability [45]. Figure 7 presents the response curves of the photodegradation rate (P) of free active Avm and Avm@SC-PLA nanocarrier systems with different ratios as a function of irradiation time. Experimental data clearly demonstrate that the photodegradation of unencapsulated active Avm proceeds extremely rapidly. Under continuous UV irradiation for 75 h, the degradation rate of this type of Avm has exceeded 59%. In contrast, the photodegradation rate of Avm loaded in Avm@SC-PLA nanocarriers remains at only approximately 30% within the same irradiation duration. This significant difference fully confirms that the Avm@SC-PLA nanoencapsulation structure can effectively enhance the photostability of Avm. Its key role lies in the fact that SC-PLA, with its high crystallinity and compact molecular structure, significantly improves UV resistance. The nanocarriers can form a dense physical protective barrier around the active Avm component, thereby blocking the destructive effect of UV light on its molecular structure and delaying the degradation process [46,47,48]. This result indicates that under UV irradiation, the outer layer of the particles can absorb, scatter, and reflect UV light, thereby providing a shielding effect for the internal Avm molecules.

### 3.5. Stability of Avermectin (Avm) Nanoformulation

**Figure 8:**
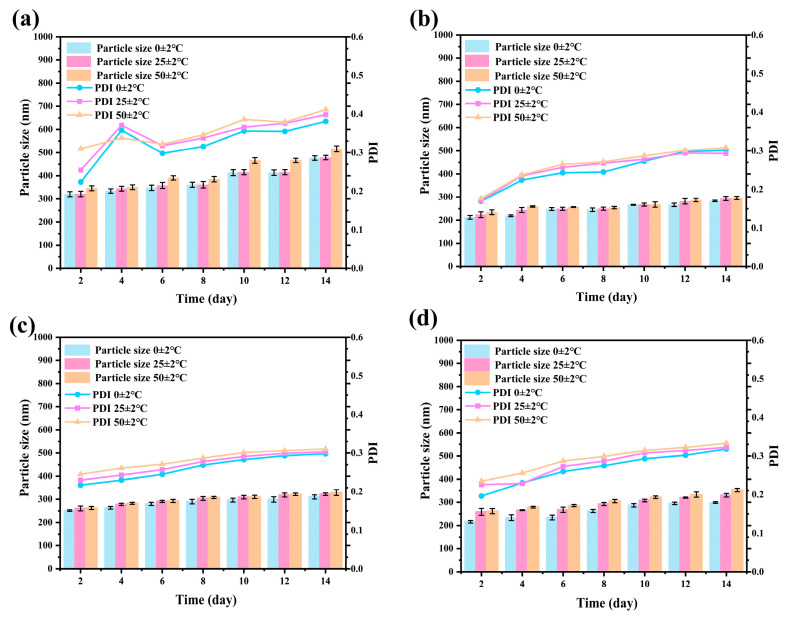
Changes in Z-average particle size and PDI of Avm@SC-PLA during storage at 0 ± 2 °C, 25 ± 2 °C, and 50 ± 2 °C. (**a**) Avm@SC-PLA 10:0, (**b**) Avm@SC-PLA 1:9, (**c**) Avm@SC-PLA 3:7, and (**d**) Avm@SC-PLA 7:3.

The storage stability of pesticide nanoformulations is a key factor determining their long-term application value [49,50]. During the 14-day storage period, under low-temperature conditions, the particle size distribution and PDI values of the nanoparticles remained constant, confirming the high structural stability of the system in refrigerated environments (Figure S2). At room temperature (25 °C), the fluctuations in particle size and PDI were maintained below 5% over 14 days, providing reliable assurance for conventional storage and transportation conditions. In the high-temperature accelerated (50 °C) test, the structural integrity of the Avm@SC-PLA system was significantly superior to that of the nanoparticle drug-loading system with a mass ratio of PLLA to PDLA of 10:0, and it exhibited notably smaller variations in particle size and PDI (Figure 8). This is mainly attributed to the high crystallinity and compact molecular structure of the SC-PLA material, which effectively inhibits thermal motion. Such high tolerance to temperature changes further verifies the application potential of the Avm@SC-PLA nanocarrier system under complex environmental conditions, laying a reliable foundation for the long-term storage and practical application of Avm.

### 3.6. Intelligent Release Behavior of Avm@SC-PLA Nanodrugs

**Figure 9:**
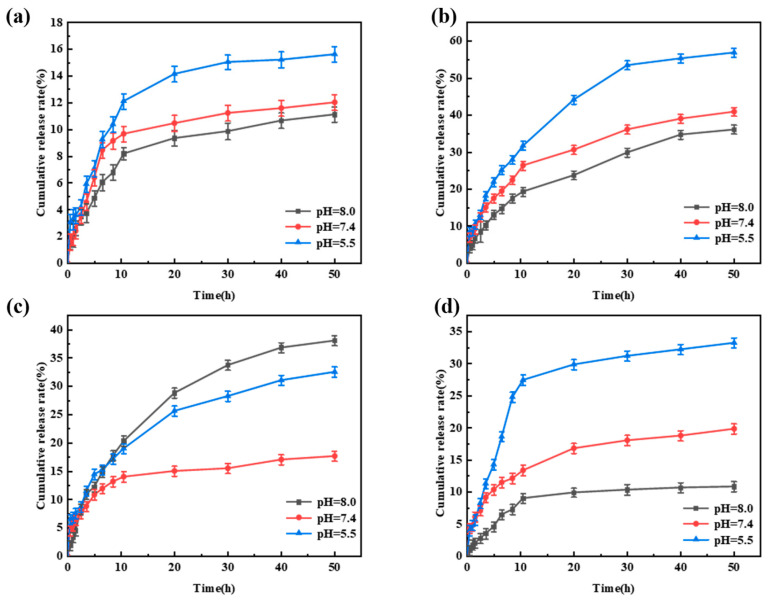
Release behavior of Avm@SC-PLA nanodrug at pH 5.5, 7.4, and 8.0. (**a**) Avm release curves of Avm@SC-PLA 10:0. (**b**) Avm release curves of Avm@SC-PLA 7:3. (**c**) Avm release curves of Avm@SC-PLA 3:7. (**d**) Avm release curves of Avm@SC-PLA 1:9.

The pH-responsive release behavior of Avm@SC-PLA drug-loaded nanospheres was evaluated through controlled release experiments under different pH levels (Figure S3) [51]. All data were statistically analyzed using SPSS software, and there were statistically significant differences among the groups (*p* < 0.05). When the system pH values were 5.5, 7.4, and 8.0, respectively, the cumulative release rates of Avm from the Avm@SC-PLA nanocarrier system with a mass ratio of PLLA to PDLA of 10:0 were 15.6%, 12.1%, and 11.2% in sequence (Figure 9a). For the Avm@SC-PLA nanocarrier system with a 1:9 mass ratio, the cumulative Avm release rates were 33.3%, 19.8%, and 10.8% in sequence (Figure 9d). The experimental results indicated that the drug-loaded nanospheres exhibited a significantly faster Avm release rate in acidic solutions. Notably, although the nanospheres with a 10:0 mass ratio showed the highest drug encapsulation efficiency, their drug release rate was extremely slow. This is because the closed and dense surface structure of the nanospheres greatly hinders the diffusion process of the active ingredients into the external environment [52]. Additionally, the relatively low specific surface area and high molecular weight of the PLLA in the carrier result in a slower hydrolysis rate in aqueous solutions, thereby delaying the Avm release process. The nanospheres with a mass ratio of PLLA to PDLA of 3:7 exhibited a fast and stable release profile within the first 10 h due to their relatively high surface roughness, with a rapid initial release rate (Figure 9c). This is attributed to the larger contact area between the nanospheres and the medium, which promotes the initial degradation of amorphous PLA. For the Avm@SC-PLA drug-loaded nanospheres with a 7:3 mass ratio, they showed the fastest release rate. This is because the cracks on their surface significantly accelerate the diffusion and escape of the drug (Figure 9b). Nevertheless, the release amount of Avm from this system within the first 3 h did not exceed 10%, indicating that it can effectively inhibit the initial burst release of the drug and avoid the waste of active ingredients.

## 4. Conclusions

This report introduces the development of a pH and temperature-responsive drug delivery system (Avm@SC-PLA). By adjusting the ratio of PLLA to PDLA and optimizing the preparation process, Avm@SC-PLA microsphere nanoformulations with different ratios and two particle size distributions (121–298 nm and 320–430 nm) were successfully prepared. The effective synthesis and uniform particle size of the Avm@SC-PLA nanospheres with different ratios were verified via multiple characterization techniques, including FTIR, UV-vis, and DLS. The Avm@SC-PLA nanospheres with different PDLA:PLLA ratios exhibited outstanding stability. During the 14-day accelerated storage experiment under thermal cycling conditions (0 °C, 25 °C, 50 °C), the particle size distribution remained basically unchanged, laying a solid foundation for the long-term storage and field application of the Avm@SC-PLA nanospheres with different ratios. They demonstrated excellent storage stability and continuous sustained-release behavior. The photosensitive Avm loaded in the Avm@SC-PLA nanospheres was protected from UV irradiation, resulting in a low degradation rate over 75 h. Within a 300 s test period, the contact angle of the Avm@SC-PLA nano-drug decreased to 49.13 ± 0.2°, and the high retention rate of the nano-drug-loaded particles on leaves indicated that these nanospheres could significantly improve the retention effect of pesticides on plant surfaces. Additionally, the Avm@SC-PLA nanospheres with different PLLA:PDLA ratios showed excellent controlled-release performance and responsiveness. Within 50 h, the cumulative release amount of Avm from the Avm@SC-PLA nanospheres with different ratios reached 56.8%. The Avm@SC-PLA nanospheres with different ratios had a reduced release rate, exhibiting typical controlled-release characteristics; moreover, their release rates increased significantly under different pH conditions. Therefore, by combining Avm@SC-PLA nanospheres with different ratios, the drug release rate can be flexibly adjusted according to the requirements of actual application scenarios, achieving precise matching of drug efficacy. In conclusion, the drug release behavior of the Avm@SC-PLA drug-loaded nanospheres can be precisely regulated by adjusting the ratio of PLLA to PDLA.

## Data Availability

The original contributions presented in this study are included in the article/Supplementary Material. Further inquiries can be directed to the corresponding author.

## Supplementary Materials

The following supporting information can be downloaded at https://www.mdpi.com/article/10.3390/ma19030492/s1, Figure S1: SEM images of (a) Avm@SC-PLA 10:0, (b) Avm@SC-PLA 7:3, (c) Avm@SC-PLA 3:7, and (d) Avm@SC-PLA 1:9; Figure S2: Visual characteristics of Avm stored for 30 days in nano-solutions with different mass ratios of PLLA to PDLA (1:9, 3:7, 7:3, 10:0); Figure S3: Fitting the release curve of Avm@SC-PLA using a kinetic model: (a)Avm@SC-PLA 10:0. (b) Avm@SC-PLA 7:3. (c) Avm@SC-PLA 3:7. (d)Avm@SC-PLA 1:9. Table S1: Thermal properties of the PLLA/PDLA nanospheres; Table S2: Drug loading content and drug loading efficiency of Avm-loaded SC-PLA nanospheres.
