# Supplementary material for: Design of Multifunctional SC-PLA Pesticide Carrier System and Study of Controlled-Release Performance
Source: Materials (Basel). 2026 Jan 26;19(3):492. doi: 10.3390/ma19030492 (PMC12898492; doi:10.3390/ma19030492)
Supplement: Supplementary file 1 [file materials-19-00492-s001.zip › materials-4090614-supplementary.pdf]

Supplementary Materials

1. Experimental section

Preparation of Avm@SC-PLA Microspheres.

First, when preparing Avm-loaded SC-PLA microspheres via the high-speed shearing method, a fixed amount (2 g) of polylactic acid (PLA) was used, and the mass ratios of PLLA to PDLA in the PLA were adjusted to four groups: 10:0, 1:9, 7:3, and 3:7. PLLA, PDLA, and a specific mass of Avm were weighed according to the above different proportions and dissolved in 50 ml of dichloromethane (DCM) with a concentration of 5% w/v. Subsequently, this organic phase was transferred into an aqueous solution of polyvinyl alcohol (PVA) with a concentration of 1.0% w/v, and mixed using a high-speed shearing machine at a rotational speed of 800 rpm. Continuous magnetic stirring was performed at room temperature for 6 hours. After complete evaporation of DCM, the microspheres were collected and centrifuged at 9500 rpm for 10 minutes. The precipitated microspheres were washed with deionized water to remove residual DCM, and this centrifugation-washing process was repeated three times. Finally, the treated microspheres were collected after freeze-drying for subsequent characterization.

2. Supplementary Figures and Tables

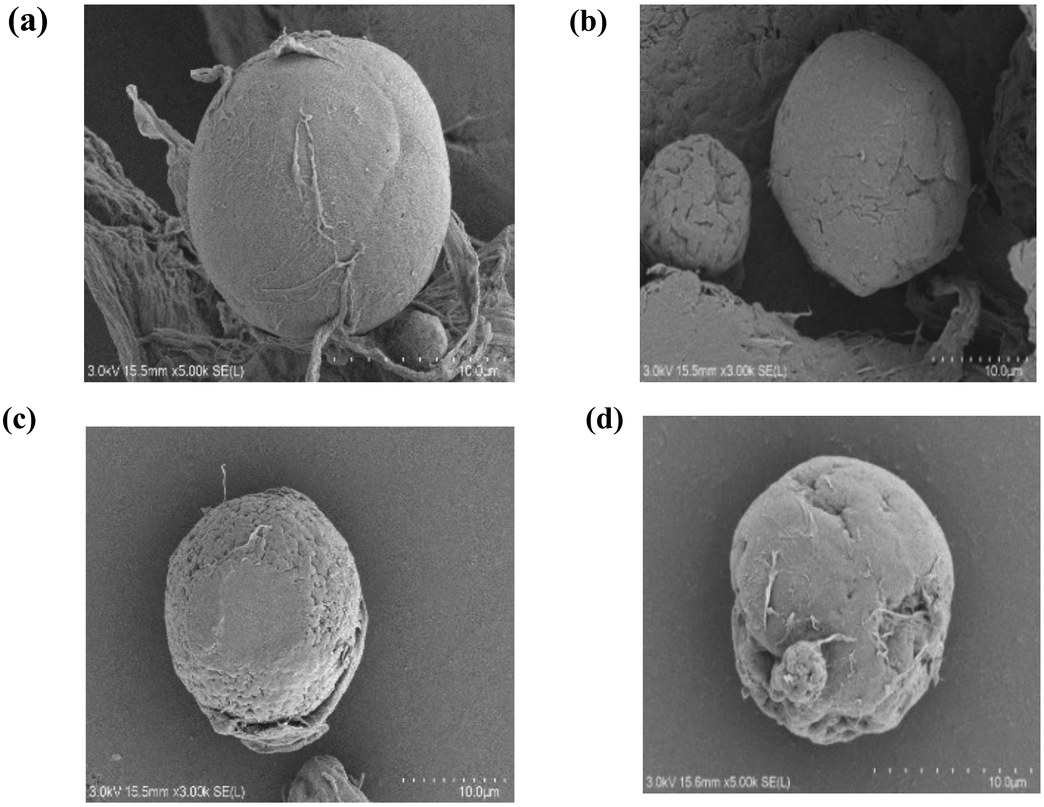

Figure S1. SEM images of (A) Avm@SC-PLA 10:0 (B) Avm@SC-PLA 7:3 (C) Avm@SC-PLA 3:7 and (D) Avm@SC-PLA 1:9

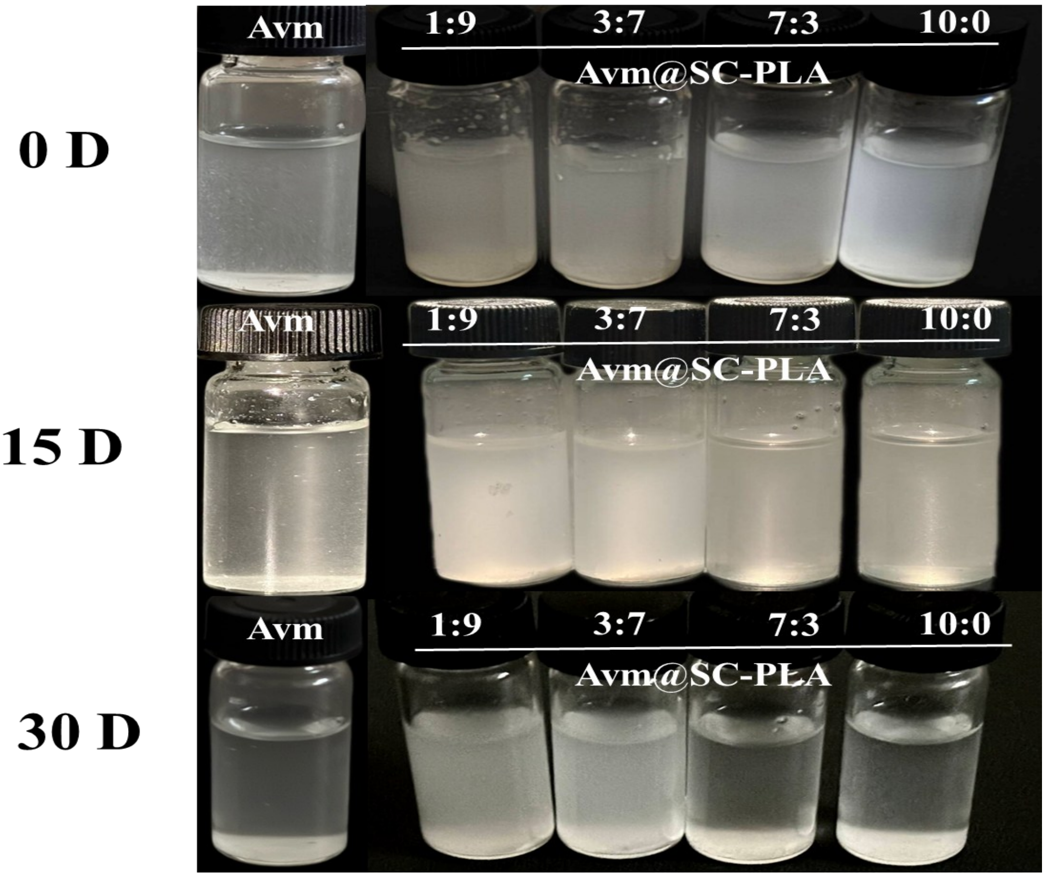

Figure S2. Visual characteristics of Avm stored for 30 days in nano-solutions with different mass ratios of PLLA to PDLA (1:9, 3:7, 7:3, 10:0)

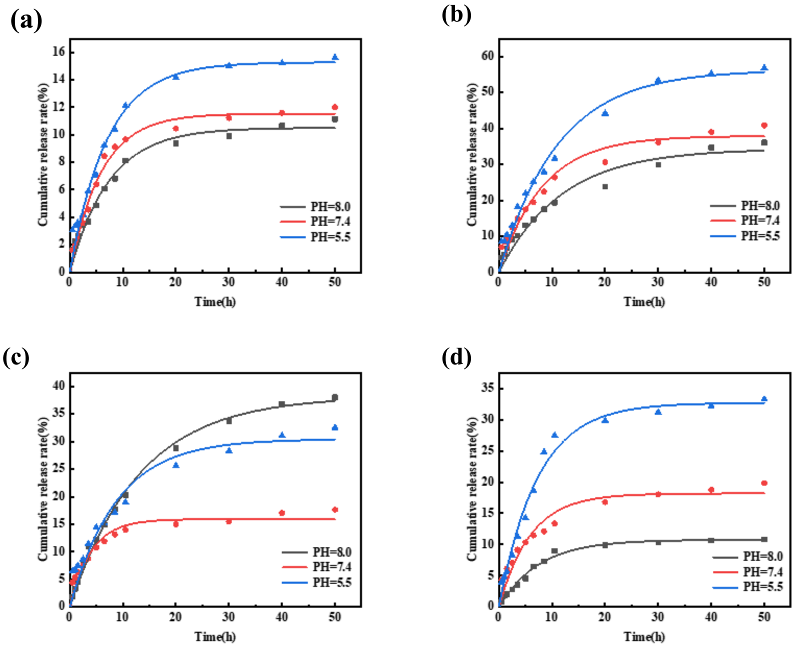

Figure S3. Fitting the release curve of Avm@SC-PLA using a kinetic model: (a)Avm@SC-PLA 10:0. (b) Avm@SC-PLA 7:3. (c) Avm@SC-PLA 3:7. (d)Avm@SC-PLA 1:9.

Table S1. Thermal Properties of the PLLA/PDLA microspheres.

Samples	T_m-L (℃)	ΔH (J/g)	Xc (%)	T_m-D (%)	ΔH (J/g)	X_c (%)	T_m-SC (℃)	ΔH (J/g)	X_c (%)
SC-PLA 10:0	161.16	29.56	31.89	/	/		/	/	/
SC-PLA 7:3	122	13.04	14.02	/	/		208.5	-65.01	45.78
SC-PLA 3:7	/	/		171.04	-25.77	27.71	218.59	-31.34	22.07
SC-PLA 1:9	/	/		170.68	-43.62	46.90	207.18	-13.77	9.69

Table S2. Drug loading content and drug loading efficiency of Avm-loaded SC-PLA microspheres.

Sample	Loading Capacity (%)	Encapsulation Efficiency
Avm@SC-PLA 10:0	19.5	80.49
Avm@SC-PLA 7:3	14.18	58.23
Avm@SC-PLA 3:7	14.11	56.74
Avm@SC-PLA 1:9	16.72	66.58
